# The transmission of *Leishmania infantum chagasi *by the bite of the *Lutzomyia longipalpis *to two different vertebrates

**DOI:** 10.1186/1756-3305-5-20

**Published:** 2012-01-19

**Authors:** Nagila FC Secundino, Vanessa C de Freitas, Carolina C Monteiro, Ana-Clara AM Pires, Bruna A David, Paulo FP Pimenta

**Affiliations:** 1 Laboratory of Medical Entomology of the Centro de Pesquisas René Rachou, Fundação Oswaldo Cruz - MG. Av. Augusto Lima, 1715 Barro Preto, Belo Horizonte, 30190-002, Minas Gerais, Brazil

**Keywords:** transmission, bite, leishmaniasis, *Lutzomyia longipalpis*

## Abstract

**Background:**

Sandflies are vectors of *Leishmania*, the causative agent of leishmaniasis in mammalian hosts, including humans. The protozoan parasite is transmitted by the sandfly bite during salivation that occurs at the moment of blood feeding. The components of vector saliva include anticlotting and vasodilatory factors that facilitate blood flow and immunomodulatory factors that inhibit wound healing and quell the immune response. Not surprisingly, these factors also play important roles in the establishment of *Leishmania *infection. To date, the majority of knowledge that has been generated regarding the process of *Leishmania *infection, including *L. infantum chagasi *transmission has been gathered by using intradermal or subcutaneous inoculation of purified parasites.

**Findings:**

This study presents the establishment of a transmission model of *Leishmania infantum chagasi *by the bite of *Lutzomyia longipalpis*, the vector of American visceral leishmaniasis. The parasites were successfully transmitted by infected sandfly bites to mice and hamsters, indicating that both animals are good experimental models. The *L. infantum chagasi *dose that was transmitted in each single bite ranged from 10 to 10, 000 parasites, but 75% of the sandflies transmitted less than 300 parasites.

**Conclusions:**

The strategy for initiating infection by sandfly bite of experimental animals facilitates future investigations into the complex and dynamic mechanisms of visceral leishmaniasis. It is important to elucidate the transmission mechanism of vector bites. This model represents a useful tool to study *L. infantum chagasi *infection transmitted by the vector.

## Findings

Despite the fact that *Leishmania *is transmitted exclusively by sandfly vectors, a reproducible animal model of *Leishmania *infection transmitted by sandfly bite was only described in the year 2000 [1].

Early investigations showed that infected *P. papatasi *sandflies can release from 0 to over 1,000 *L. major *promastigotes through their proboscides by forced feeding [2]. However, a transmission model by bite with *P. duboscqui *infected with *L. major *found that the parasite numbers inoculated in the host skin by one insect vector alone could vary from 10 to 100,000 [3].

Considering the New World species of *Leishmania*, using a feeding device of chick skin membrane over culture medium showed that *L. longipalpis *sandflies infected with *Leishmania mexicana *(an unnatural vector/parasite pair) expelled an average of 1,000 parasites per fly [4]. Thus, even though *L. longipalpis *is the vector of *L*. *infantum chagasi *in nature, it is permissive in the laboratory to infection with other Leishmania species.

In consideration of the continuing prevalence of American visceral leishmaniasis and the paucity of related studies in the literature, our goal was to develop an *L. infantum chagasi *transmission model by bite, using its proven vector, *L. longipalpis*. In addition, we aimed to define the amount of parasites that are directly expelled into the mammalian host, which has never before been carried out.

To evaluate the sandfly infection, colonized two- to four-day-old *L. longipalpis *females (Lapinha Cave strain) were infected by feeding through a chick skin membrane (feeding device) on mouse blood containing 4×10^6^/mL *L. infantum chagasi *(MHOM/BR/70/BH46) promastigotes. After two, six, nine and fourteen days, the midguts of infected flies were dissected and the parasite load was estimated by hemocytometer counting.

**Figure 1:**
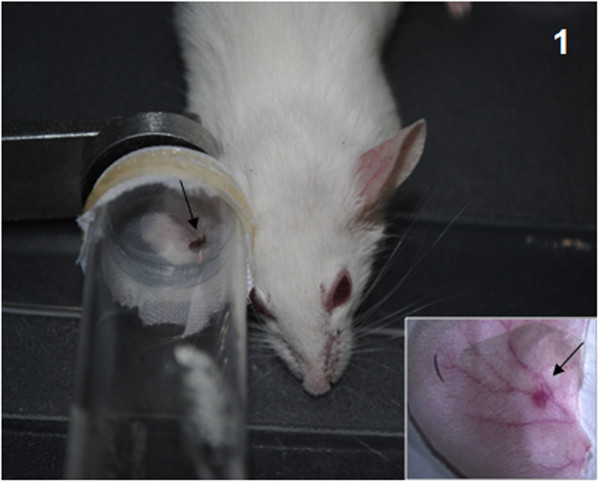
***L. longipalpis *sandfly biting a Balb/C mouse**. A single sandfly was confined within a vial and allowed to bite the animal's entire ear. Note the blood engorgement of the sandfly (arrow). **Inset**: Dissected mouse ear showing the bite site as a small red dot (arrow).

For Transmission by bite, fourteen-day infected flies were transferred to small plastic vials (3-dram volume, 4.8-cm height, 1.8-cm diameter) covered at one end with a 0.25-mm nylon mesh. Balb/C mice and hamsters were anesthetized by Thiopental injection. Clamps were used to hold the mesh end of each vial flat against the animals' ears so that the fly had access to the ear skin for feeding over a period of 1-2 h in the dark. The animals were then euthanized and the exposed ears were dissected for testing the parasite presence. One infected sandfly was used for each of the transmission experiments (Figure 1). All animals were maintained at the Animal Care Facility of the FIOCRUZ-MG under specific pathogen-free conditions and were used in accordance to a study protocol approved by the FIOCRUZ Ethical Committee for Animal Use (CEUA; license number LW30/10).

Exposed-bite animal ears were processed for DNA extraction and real-time PCR as described elsewhere [3]. Parasite quantification was performed by automatic comparison with the specific set of standard samples prepared in parallel to each set of test samples. The number of *L. infantum chagasi *in each sample was summarized as the mean of the two median values from the three reactions in each run. Twenty-eight or 30 ears from each group (mouse or hamster) were processed.

**Figure 2:**
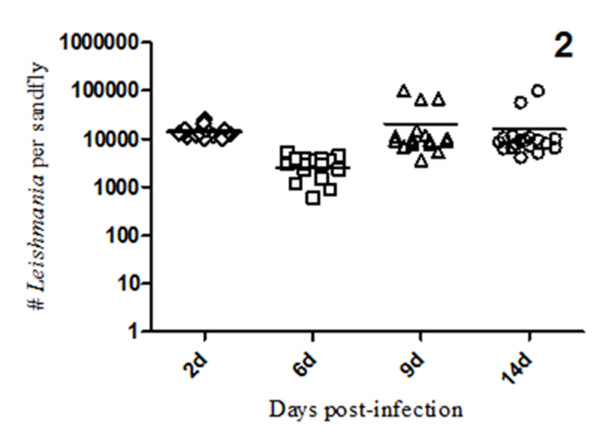
**Parasite numbers in infected *L. longipalpis *(2 to 14 days after the infective bloodmeal)**. Flies were infected by membrane feeding on mouse blood containing 4 million logarithmic phase promastigotes/mL. Midguts were dissected on the indicated days post-feeding and scored for numbers of viable parasites.

A total of 640 *L. longipalpis *females from 800 individuals ingested the infected bloodmeal containing *L. infantum chagasi *promastigotes. A group of 300 sandflies from this collection were dissected and evaluated up to 14 days after infection. Seventy-two hours after the infective bloodmeal, 100% of the sandflies were positive for viable promastigotes, with an average of 14,000 promastigotes per midgut. At six days post-infection, there was an approximate 70% reduction in parasite growth and survival (*p *= 0.0009), with an infection average of 3, 000 parasites per sandfly; this change may reflect loss of parasites due to fly defecation or the hostile digestion process, as described previously [5]. At 9 to 14 days post-infection, the observed *L. infantum chagasi *growth inside the sandfly indicated that a mature infection had been established reaching an average of 10,000 promastigotes/midgut (Figure 2).

**Figure 3:**
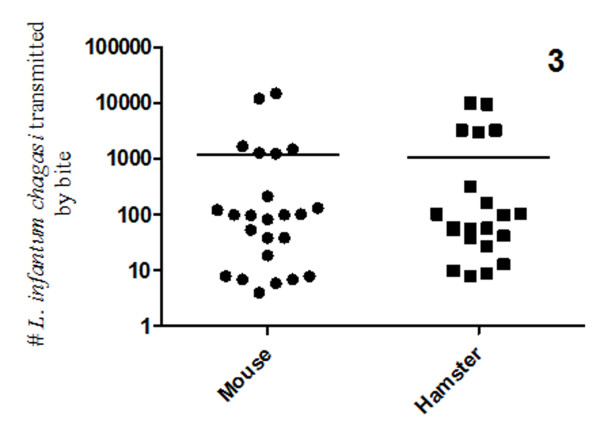
***L. infantum chagasi *transmitted to the mouse and hamster ears**. The parasite numbers were obtained by real-time PCR of dissected ears at two hours after the bites. No significant differences were observed for parasite transmission by the various sandflies.

To develop a transmission model for laboratory use, we initially used two groups of animals: mice and hamsters. Twenty-eight ears of each animal group were exposed to a single infected *L. longipalpis*. The detection and quantification of *L. infantum chagasi *transmitted by the sandfly bites were carried out using real-time PCR. The parasite doses expelled from the sandflies to the mouse ears ranged from 4 to 15,000 (mean: 1,255), and to hamster ears ranged from 8 to 10,000 (mean: 1,122). There was no significant difference in the parasite number expelled by *L. longipalpis *bites into mice or hamsters, suggesting each can be used as model for transmission of *L. infantum chagasi *by its infected sandfly vector (Figure 3).

**Figure 4:**
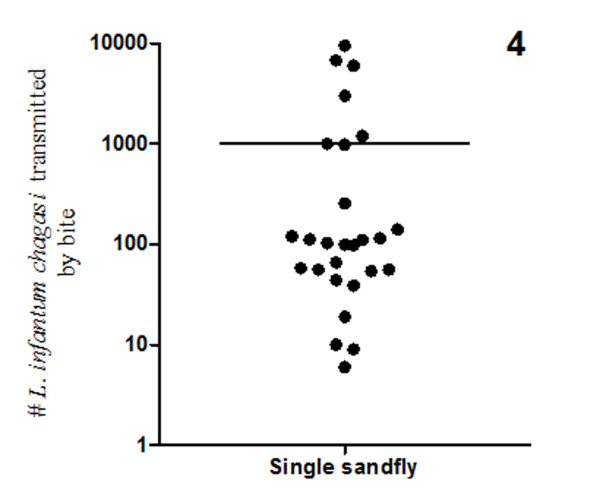
***L. infantum chagasi *transmitted to the mouse ears by a single infected *L. longipalpis***. Note that although the main number of transmitted parasites was about 1,000 the majority of the ears presented < 300 *L. infantum chagasi*.

When only a single sandfly was allowed to bite the mouse ears (n = 30 ears), real-time PCR detected the presence of *L. infantum chagasi *in 27 ears. The three that were negative for PCR detection of parasites also did not show outward signs of bites. In the infected ears, the parasite quantification showed that transmitted dose by the bite of a single *L. longipalpis *ranged from 10 to 10, 000 (mean: 1, 002). Finally, it was determined that 75% of the sandflies transmitted < 300 *L. infantum chagasi *in each ear (Figure 4).

In the New World, particularly in Brazil, leishmaniasis has followed the massive migration of people from rural areas into urbanized regions. In addition, the density of metropolitan areas and ease of travel between the two areas has led to increasing incidence rates of this vector-transmitted infectious disease [6]. Studies with the Brazilian vector species are, thus, necessary to understand the underlying mechanisms of parasites, vector, and the vertebrate hosts, as well as the complex interactions between the three, which support disease onset and persistence.

In order to establish, for the first time, a transmission model of laboratory infection of visceral leishmaniasis, we used *L. longipalpis *infected with *L. infantum chagasi *to initiate infection by bite in laboratory animals. This approach probably is superior to the traditional needle inoculation method, which eliminates the natural transmission features (saliva, natural doses expelled through the proboscides).

Initially, we evaluated and compared hamsters and Balb/C mice as mammalian hosts for experimental infection. We concluded that there is no significant difference between these two animals in their utility as a model for transmission of visceral leishmaniasis by the vector bite. The two animals were bitten equally and the parasite numbers expelled into the ears by the *L. longipalpis *sandfly were similar, demonstrating that they are effective mammalian hosts for transmission of laboratorial infection.

Our results showed that the amount of *L. infantum chagasi *promastigotes delivered by a single *L. longipalpis *to the ears ranged from 4 to 10, 000 (mean: 1, 002). Coincidently, these numbers are similar to the findings from the force-feeding study [2] and others, in which *L. longipalpis *were artificially infected with *L. mexicana *and then forced to expel parasites into culture medium [4].

Interestingly, it is worth noting that in an experimental study of Old World cutaneous leishmaniasis the amount of *L. major *injected into mouse skin by a single sandfly, *P. duboscqui *could reach 100,000 parasites, but the majority of sandflies transmitted ~600 parasites [3].

Collectively, with our analysis, these findings indicated that the New World sandfly *L. longipalpis *cannot transmit as many parasites as the Old World sandfly. The majority of parasite loads transmitted by *L. longipalpis *are very low, since 75% of them transmitted < 300 parasites in the laboratory conditions. Only a few flies transmitted around 10, 000 parasites, a number that is still 10-fold less than that of the *P. duboscqui*. Further studies using other vector sandfly species are necessary to better understand this difference.

This study provided a strategy for initiating infection of experimental animals by vector bite that will facilitate future investigations into the complex and dynamic mechanisms of visceral leishmaniasis. The use of this model of parasite transmission may help to develop new strategies for prevention and treatment of leishmaniasis, a human life-treating disease.

## Competing interests

The authors declare that they have no competing interests.

## Authors' contributions

NFCS conceived and designed the experiments; NFCS and PFPP analyzed the data and wrote the paper; VCF, CCM, ACAMP, BAD performed the experiments. All authors read and approved the final version of the manuscript.
